# The Book World of Medicine and Science

**Published:** 1907-08-10

**Authors:** 


### The Book World of Medicine and Science.

A System of Medicine by Eminent Authorities in Great Britain, the United States, and the Continent. Edited by Wm. Osler, M.D., F.R.S., assisted by Thomas McCrae, M.D., F.R.C.P. Volume I. (Henry Frowde, Oxford University Press; Hodder and Stoughton, London, 1907.)

To organise and conduct a "System of Medicine" is an undertaking of no little responsibility. Probably no one but the actual editor of such a publication can have more than a vague and partial appreciation of the delicacy and difficulty of the task. Wide and catholic sympathies, an impartial sense of proportion, contact with all modern advances, an accurate judgment of personal and professional values, and a capacity for organisation and control, are among the elementary equipment which is essential if success is to crown the work. In addition, how shall one picture the constant patience and tact necessary to meet and overcome the recurring and various difficulties which must inevitably arise in the effort to secure adherence to the spirit and method of the original plan? Contributors, even with the best will in the world, are apt to prefer their own point of view, and yet, too lax a control may readily wreck the symmetry and value of the entire scheme. It is considerations such as these which, more or less consciously, make all of us disposed at the very outset to look, in connection with any such enterprise, for the name of the editor. This legitimately anxious curiosity will, we are sure, on the present occasion be more than abundantly satisfied. For if there is anyone to whose editorial capacity and judgment professional confidence will be readily and freely extended, it surely is the Regius Professor of Medicine in the University of Oxford. In a word, the new "System of Medicine," under the guidance of Professor Osler, enters on its career under the best and most fortunate auspices.

The first part of the "System," which now lies before us, is a substantial volume of some nine hundred pages. It deals with a number of different subjects, and we do not doubt that the goodwill which is due from the whole profession to the editor, will also be extended to his contributors. Adequately to review such a volume in the space at our disposal is manifestly impossible. We can only hope to give an outline of the general character of the work, and to notice briefly one or two of the more important contributions it contains. Perhaps it would be fair to say that one of the most prominent features of the articles is the attempt to associate the practical application of medicine at the bedside with the scientific knowledge and methods of the present day. There is, and probably for long there must be, much in medical practice which is both empirical and arbitrary. But the range of the rational and scientific is always increasing, and to fit the advance in these directions to clinical work is a great and worthy enterprise. Especially is such an attempt to be commended when it aims to reach not merely the consulting and hospital physician, but also those who carry the beneficent work of medicine into the lives and homes of the people. Professor Osler, in various ways, has shown so sympathetic an appreciation of the difficulties and opportunities of the general practitioner, that it is no surprise to find this tone and spirit in any enterprise which enjoys his direction. We are sure, that if preserved throughout, as it doubtless will be, this will be one of the features of the work to which cordial and appreciative recognition will be extended.

Turning now to individual articles, we have first an "Introduction" by the editor entitled "The Evolution of Internal Medicine." This has the graceful and virile qualities to which we are accustomed in Professor Osler's writings, and it is, at the same time, a lucid and succinct sketch of the historical movement in medicine, together with some suggestions of the direction in which further progress is to be anticipated. Following this is a chapter on "Inheritance and Disease" by Professor J. G. Adami; it is well worthy of his reputation, and is certainly one of the most interesting articles in the volume. The complexities of the subject are most lucidly explained, and the more recent views on the question are amply discussed. The second section of the work is occupied by diseases caused by physical agents, and here the effects capable of being produced by heat, light, electricity, and the *x*-rays receive recognition. The poisonous actions of various inorganic substances, such as mercury, lead, arsenic, etc., are next considered; whilst alcohol, opium, cocaine, and the food poisons, occupy the succeeding chapter.

Chapter XIII. is devoted to "Auto-intoxications," and is by Dr. A. E. Taylor, Professor of Pathology in the University of California. It is well worthy of special mention, and the study of it may help to banish some of the loose thinking and talking which have gathered round this subject. This article, more especially when considered in conjunction with a later one on "Metabolism" by Professor Chittenden, is a forcible illustration of the progress that has been made in our knowledge of physiological chemistry, and of the wide range which this knowledge covers in relation to the nature of the changes which occur in disease. If pathological anatomy is an almost exhausted mine, pathology, as a study of processes, yet offers large tracts for exploration, and many of these cannot even be entered upon until the details of normal metabolism have been defined. The present volume is particularly well equipped as a statement of the present state of science in these various respects, and the articles mentioned above may be heartily commended to all practitioners anxious to supply themselves with the most recent scientific information and with the application of this to medical diagnosis and treatment.

Diseases caused by protozoa and the animal parasites, which come next in order, form a very successful section, and special attention has been paid to the illustration and accurate description of the organisms concerned. In the concluding section, Dr. Chittenden writes on "Metabolism," and Dr. Futcher on "Diabetes" and on "Gout," while Dr. Still contributes a chapter on "Rickets," and Dr. Robert Hutchison one on "Scurvy." Altogether this new "System of Medicine" has made an excellent start, and is sure of a wide field of usefulness. The comment has been made that the list of contributors includes the names of comparatively few British writers, whilst Transatlantic medicine is strongly represented. Even though this be admitted, we by no means accept the suggestion that such an arrangement will make the work less acceptable to readers in this country. Obviously there is an advantage in having facts presented and discussed from a somewhat different point of view from the one to which we are accustomed, and the note of freshness which characterises most of the articles is one of the most pleasing features of the present volume. The subjects included under "Internal Medicine" are for the most part so complex and so obscure that there is abundant room for the play of individuality. Hence, without in the least questioning the value of other similar works, and entirely abstaining from invidious comparisons, we are convinced that there is abundant room for the "System of Medicine" which has the good fortune to enjoy the inspiriting direction of Professor Osler and Dr. McCrae.

